# Iliac fossa vs Pfannenstiel retrieval incision in laparoscopic donor nephrectomy: A critical analysis

**DOI:** 10.1080/2090598X.2019.1637069

**Published:** 2019-07-22

**Authors:** Chaitanya S. Deshmukh, Arvind P. Ganpule, S. Balaji Sudharsan, Abhishek G. Singh, Ravindra B. Sabnis, Mahesh R. Desai

**Affiliations:** Department of Urology, Muljibhai Patel Urological Hospital, Nadiad, India

**Keywords:** Pfannenstiel, Iliac fossa, laparoscopy, donor nephrectomy

## Abstract

**Objective**: To compare two retrieval incisions, Pfannenstiel vs iliac fossa incision, in terms of operative technique-related variables and variables related to patient satisfaction postoperatively, in patients undergoing laparoscopic donor nephrectomy (LDN).

**Patients and methods**: This prospective randomised study was conducted between May 2016 and April 2017. All the voluntary kidney donors aged 18–60 years were randomised into two groups. Group 1, comprised patients undergoing graft retrieval via an iliac fossa incision, and Group 2 comprised those undergoing graft retrieval via a Pfannenstiel incision. Intraoperative assessment of the incision by the surgeon was done using a Likert scale-based questionnaire. Other variables studied were the operative time, retrieval time, warm ischaemia time, and length of incision. Postoperatively, visual analogue scale pain scores, analgesia consumption, and hospital stay were compared. During follow-up cosmetic outcome was compared.

**Results**: In all, 108 patients were enrolled in the study with 54 patients in each group. The mean operative time was shorter in Pfannenstiel-incision group, at 155.2 vs 171.67 min (*P* = 0.01). The retrieval incision length was significantly less in the Pfannenstiel arm, at 9.29 vs 9.85 cm (*P *< 0.001). In the surgeon Likert scale-based questionnaire, the Pfannenstiel incision scored better than the iliac fossa incision for ease of specimen retrieval (*P* = 0.015), ease of immediate check laparoscopy (*P* = 0.002), and ease of incision closure (*P* < 0.001). The Pfannenstiel-incision group required less postoperative analgesia, at a mean (SD) of 7.03 (8.82) vs 15.55 (11.1) mg nalbuphine (*P* < 0.001). During follow-up the Manchester Scar Scores were lesser in the Pfannenstiel-incision group (*P* < 0.001).

**Conclusion**: The Pfannenstiel incision was considered preferable during the critical steps of the LDN and had a smaller retrieval incision, lesser operative time and postoperative analgesia requirement, and better cosmesis than the iliac fossa incision.

**Abbreviations:** BMI: body mass index; LDN: laparoscopic donor nephrectomy; VAS: visual analogue scale; WIT, warm ischaemia time

## Introduction

When it comes to offering a therapy for end-stage renal failure, kidney transplant is the therapy of choice [1]. The recipient can receive a kidney either from a cadaver or from a living donor. Living-donor kidney transplantation is associated with advantages such as reduced waiting-list period, elective nature of the procedure, and better graft and patient survival compared to cadaveric kidney donation [2].

Laparoscopic donor nephrectomy (LDN) is now the ‘gold standard’ and preferred method for kidney harvest in renal transplant surgery [1]. Amongst the operative steps involved in LDN, graft retrieval is one of the most critical steps. The kidney can be retrieved through a Pfannenstiel, iliac fossa, midline periumbilical, or subcostal flank incision [3].

We conducted the present study to compare two of these retrieval incisions, Pfannenstiel vs iliac fossa incision, in terms of operative technique-related variables and variables related to patient satisfaction postoperatively.

## Patients and methods

This was a single-centre, prospective, randomised, comparative study that included all the donors undergoing LDN from May 2016 to April 2017. After approval from the Institutional Ethics Committee (EC/370/2016), patients undergoing laparoscopic live-donor nephrectomy for renal transplantation were randomised into two groups using a random number table. Group 1, comprised patients undergoing graft retrieval via an iliac fossa incision and Group 2, comprised patients undergoing graft retrieval via a Pfannenstiel incision.

All voluntary kidney donors, aged 18–60 years, undergoing either right or left LDN surgery were included.

The exclusion criteria were: patients who were uncooperative and unwilling to be followed up; needed conversion to open donor nephrectomy; marginal donors [4,5]; those with a previous history of abdominal surgeries or abdominal incisions; and those in whom the site of retrieval incision changed intraoperatively.

Voluntary donors underwent LDN via a transperitoneal approach as per the institutional operative protocol [6]. For a Pfannenstiel incision the patient was placed in a lateral decubitus position, with the operating surgeon seated on a chair. At 3.5 cm (two fingerbreadths) above the pubic symphysis, a transverse skin incision was made and the superficial and deep facia were divided up to the anterior rectus sheath. The rectus sheath was incised transversely and the superior and inferior flaps were raised. The rectus muscles were split in the midline and retracted. The pre-peritoneal fat was incised but the peritoneum was left intact. The wound was packed with wet gauze.

**Figure 1.**
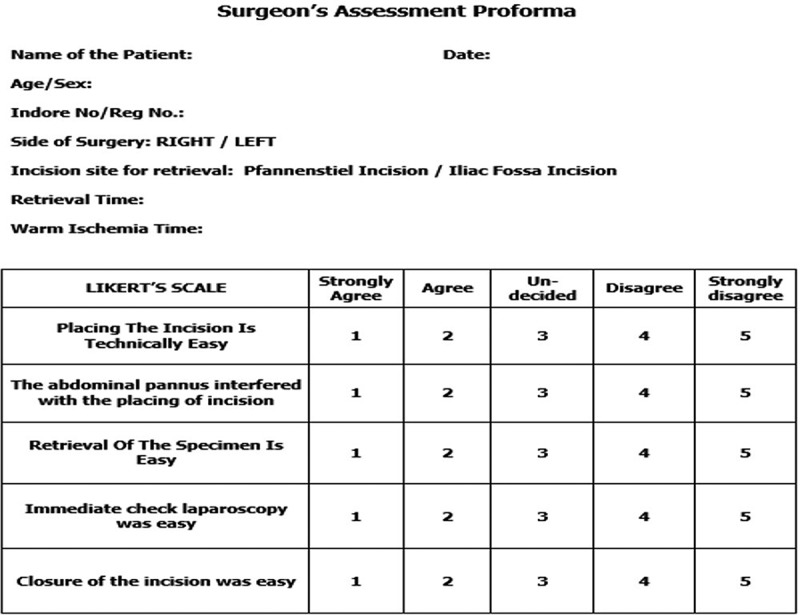
Likert scale-based surgeon’s assessment pro forma.

For iliac fossa retrieval, the incision was made with the patient in a lateral decubitus position. At 3.5 cm (two fingerbreadths) above and parallel to the inguinal crease, an oblique skin incision was made and deepened up to the external oblique aponeurosis. The incision was placed in manner so as not to include any port sites in the incision. The anterior abdominal wall muscles were cut with cautery and the peritoneum reached. The pre-peritoneal fat was incised but the peritoneum was left intact. For both the incisions, the peritoneum was incised and the abdominal cavity was entered only after the graft kidney was completely detached at the hilum. Following the completion of the LDN, the operating surgeon was given a 5-point Likert scale-based surgeon’s assessment pro forma [7]. Response to each parameter is scaled on five grades; 1, strongly agree; 2, agree; 3, undecided; 4, disagree; and 5, strongly disagree (Figure 1).

Other variables studied were the operative time, retrieval time, warm ischaemia time (WIT), and length of incision. Postoperatively, the level of pain experienced by the patient was assessed using a Universal Pain Assessment Tool [8]. The pain was assessed at specific time intervals from the completion of surgery, at 0, 2, 4, 8, 16, 24, and 48 h. Any wound complication that developed during the hospital stay was recorded. Other variables compared in the postoperative period were analgesia consumption and hospital stay.

**Figure 2.**
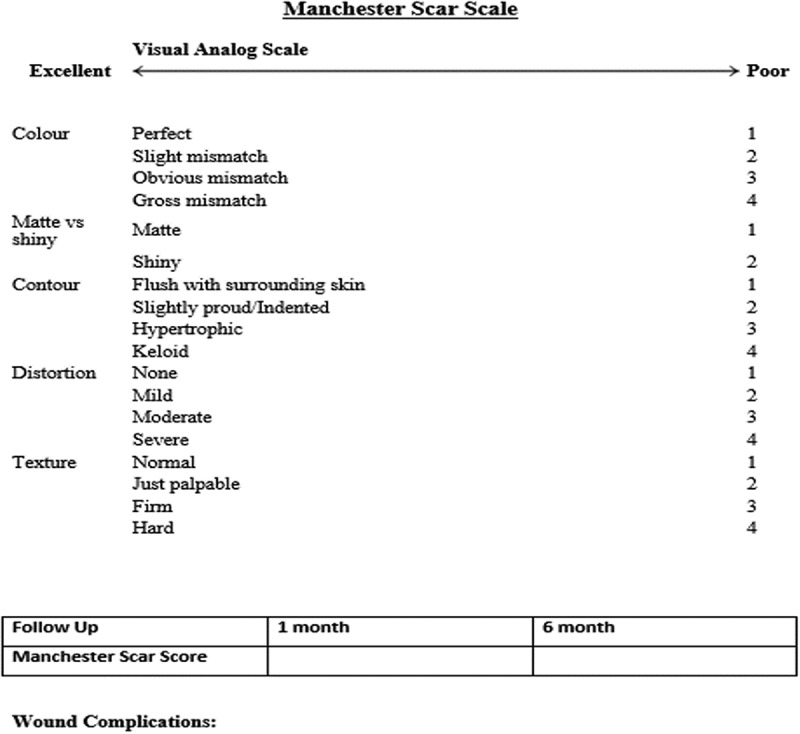
Manchester Scar Scale.

During follow-up, cosmesis related to the scar was assessed at 1 and 6 months. To assess patient satisfaction related to the operative scar at the retrieval incision site the Manchester Scar Scale [9] was used (Figure 2); the higher the score the poorer the cosmetic outcome. Any wound complications during follow-up were recorded.

### Statistics

We used the Statistical Package for the Social Sciences (SPSS®) version 15.0 software (SPSS Inc., IBM Corp., Armonk, NY, USA) for the statistical analysis. The Student’s *t*-test was used for testing the difference between the means of the study groups. We used the chi-squared test for testing the significance of categorical variables.

## Results

**Figure 3.**
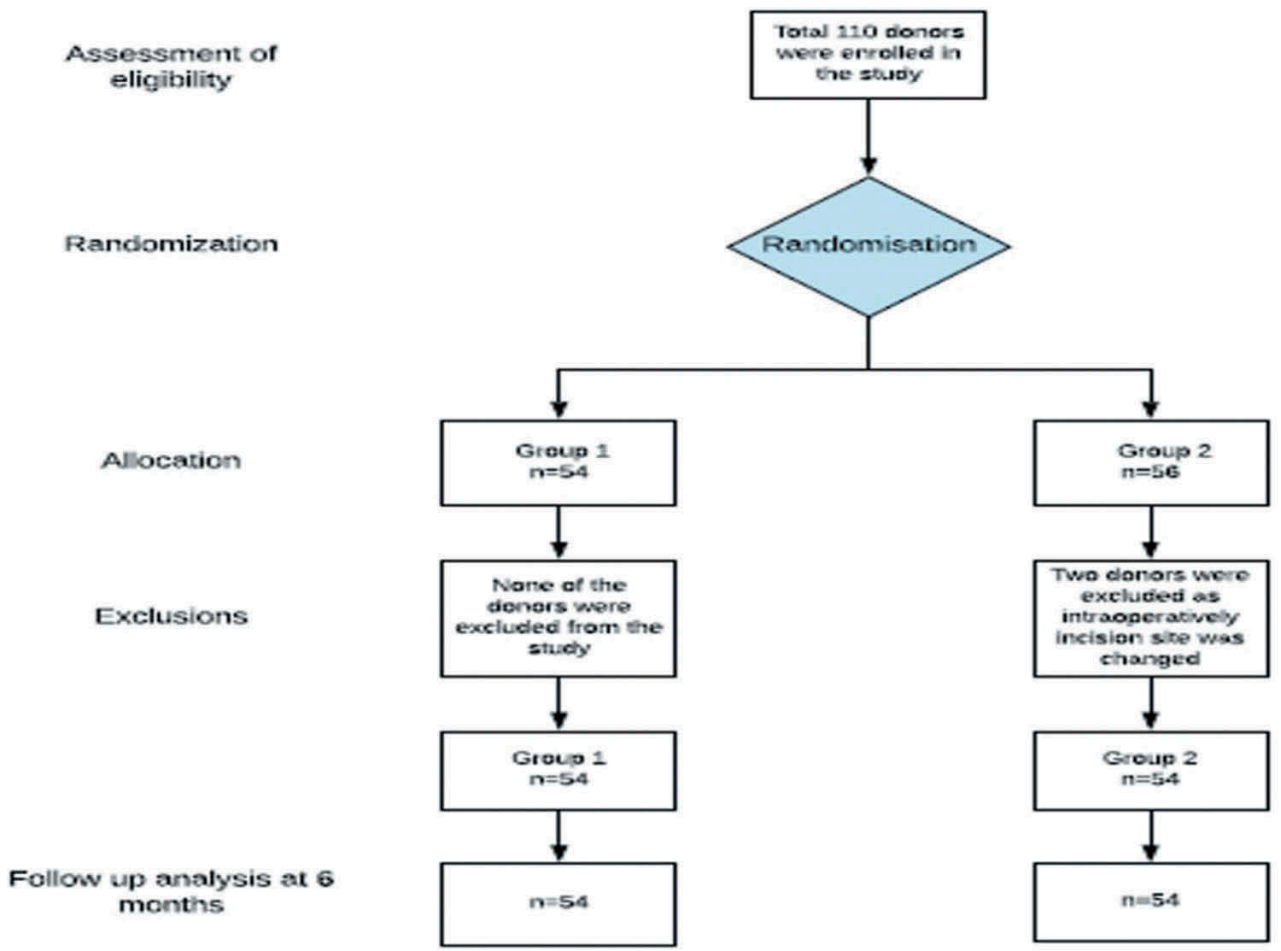
Consolidated Standards of Reporting Trials (CONSORT) diagram.

In all, 108 patients were randomised into two groups after exclusions (Figure 3). Both, the iliac fossa-incision group (Group 1) and Pfannenstiel-incision group (Group 2) consisted of 54 patients each. The preoperative demographics of the patients in both groups are summarised in Table 1. The intraoperative, postoperative, and follow-up variables are summarised in Tables 2 and 3.

**Table 1.** Preoperative demographics.

Variable	Group 1 Iliac fossa incision	Group 2 Pfannenstiel incision	*P*
Number of patients	54	54	–
Age, years, mean (SD)	46.87 (10.55)	47.91 (8.01)	0.56
Gender: M/F, *n* (%)	11 (20.4)/43 (79.6)	15 (27.8)/39 (72.2)	0.50
Left side, *n* (%)	52 (96.3)	49 (90.7)	–
Right side, *n* (%)	2 (3.7)	5 (9.3)	–
Preoperative haemoglobin, g/dL, mean (SD)	11.96 (1.17)	12.49 (1.26)	0.94
Serum creatinine, mg/dL, mean (SD)	0.66 (0.13)	0.70 (0.14)	–
BMI, kg/m^2^, mean (SD)	26.79 (4.14)	25.26 (3.55)	0.04
Comorbidities, *n*	Hypertension – 2	Hypertension – 4	–
	Hypothyroid – 1	Pre-diabetic – 1	
	Parkinson’s disease – 1	Depression – 1	
	Beta thalassemia trait – 1	Mood disorder – 1	
		Psoriasis – 1

**Table 2.** Intra- and postoperative variables.

Variable	Group 1 Iliac fossa incision	Group 2 Pfannenstiel incision	*P*
Mean (SD):			
Total operative time, min	171.67 (36.81)	155.2 (27.77)	**0.01**
Retrieval time, s	226.17 (91.92)	229.72 (27.77)	0.83
… WIT, s	310.37 (115.44)	310.65 (104.36)	0.99
Length of retrieval incision, cm	9.85 (0.32)	9.29 (0.22)	**<0.001**
Likert scale-based questionnaire:			
Ease of making the incision	2.20 (0.68)	2.22 (0.66)	0.88
Interference of abdominal pannus in making the incision	3.72 (0.76)	2.98 (1.36)	**0.001**
Ease of specimen retrieval	2.46 (0.60)	2.17 (0.63)	**0.015**
Ease of immediate check laparoscopy	2.31 (0.60)	1.89 (0.74)	**0.002**
Ease of incision closure	2.20 (0.73)	1.57 (0.79)	**<0.001**
Postoperative VAS pain score* at:			
0 h	3.13 (0.64)	3.02 (0.56)	0.34
2 h	3.37 (0.68)	3.19 (0.51)	0.11
4 h	3.24 (0.72)	3.17 (0.72)	0.59
8 h	3.14 (0.65)	2.85 (0.62)	**0.018**
16 h	2.87 (0.64)	2.44 (0.71)	**0.002**
24 h	2.19 (0.91)	1.85 (0.68)	**0.03**
48 h	0.76 (1.04)	0.74 (0.99)	0.93
Total analgesia consumption, mg nalbuphine	15.55 (11.1)	7.03 (8.82)	**<0.001**
Haemoglobin drop, g/dL	0.78 (0.6)	0.80 (0.73)	0.85
Postoperative hospital stay, days	2.23 (0.67)	2.17 (0.28)	0.43
Manchester Scar Score at:			
1 month	12.93 (0.84)	11.81 (0.8)	**<0.001**
6 months	7.83 (0.81)	6.63 (0.81)	**<0.001**

Bold *P* values significant.

**Table 3.** Likert scale-based parameters.

Parameter	Group 1 Iliac fossa incision, mean (SD)	Group 2 Pfannenstiel incision, mean (SD)	*P*
**BMI <25 kg/m^2^**			
Ease of placing the incision	1.76 (0.43)	2 (0.27)	0.059
Interference of abdominal pannus in placing the incision	4.12 (0.78)	3.85 (1.09)	0.35
Ease of specimen retrieval	2.18 (0.39)	1.89 (0.42)	**0.028**
Ease of immediate check laparoscopy	2.06 (0.65)	1.56 (0.50)	**0.012**
Ease of incision closure	1.76 (0.75)	1.19 (0.39)	**0.008**
**BMI 25–30 kg/m^2^**			
Ease of placing the incision	2.37 (0.62)	2.26 (0.65)	0.58
Interference of abdominal pannus in placing the incision	3.48 (0.75)	2.16 (0.89)	**<0.001**
Ease of specimen retrieval	2.59 (0.63)	2.32 (0.47)	0.09
Ease of immediate check laparoscopy	2.33 (0.55)	2.11 (0.56)	0.18
Ease of incision closure	2.26 (0.59)	1.79 (0.78)	**0.03**
**BMI >30 kg/m^2^**			
Ease of placing the incision	2.5 (0.85)	2.88 (1.12)	0.44
Interference of abdominal pannus in placing the incision	3.7 (0.48)	2 (1.3)	**0.007**
Ease of specimen retrieval	2.6 (0.69)	2.75 (1.03)	0.73
Ease of immediate check laparoscopy	2.7 (0.48)	2.5 (1.19)	0.66
Ease of incision closure	2.8 (0.63)	2.38 (1.06)	0.33

Bold *P* values significant.

The mean (SD) retrieval time and WIT were comparable between groups 1 and 2, at 226 (91.92) vs 229.72 (86.03) s (*P* = 0.83) and 310.37 (115.44) vs 310.65 (104.36) s (*P* = 0.99), respectively. The total operative time was less in Group 2.

The mean (SD) incision length was significantly shorter in Group 2 as compared to the Group 1, at 9.29 (0.22) vs 9.85 (0.32) cm (*P* < 0.001)

In the Likert scale-based questionnaire (completed by two surgeons for each of the 108 donor individuals), the lower the score means that the surgeon agrees with the question at hand and the higher the score means the more he disagrees with the statement. Groups 1 and 2 were comparable as far as ease of placement of the incision was concerned (*P *= 0.88). Abdominal pannus interfered in placing the incision in Group 2, with a mean (SD) Likert-scale score of 3.72 (0.76) vs 2.98 (1.36) (*P* = 0.001). Ease of specimen retrieval was assessed and here Group 2 scored better than Group 1 (*P* = 0.015). As far as the ease of immediate check laparoscopy and incision closure were concerned, Group 2 scored better than Group 1 (*P *= 0.002 and *P* < 0.001, respectively). These outcomes were also stratified according to the donor’s body mass index (BMI; Table 3). In patients with a BMI of <25 kg/m^2^, Group 2 scored better than Group 1 for ease of specimen retrieval [mean (SD) Likert-scale score of 2.18 (0.39) vs 1.89 (0.42); *P* = 0.028], ease of immediate check laparoscopy [2.06 (0.65) vs 1.56 (0.5); *P* = 0.012], and ease of incision closure [1.76 (0.75) vs 1.19 (0.39); *P* = 0.008]. Amongst patients with a BMI of 25–30 kg/m^2^, Group 1 scored more than Group 2 in terms of interference of abdominal pannus with the placement of incision [mean (SD) Likert-scale score 3.48 (0.75) vs 2.16 (0.89); *P* < 0.001], suggesting that the abdominal pannus interfered more in Group 2 patients. In this BMI category, Group 2 scored better than Group 1 for ease of incision closure [mean (SD) Likert-scale score 2.26 (0.59) vs 1.79 (0.78); *P* = 0.03). In patients with a BMI >30 kg/m^2^, interference of the abdominal pannus in making the incision was significantly different between groups 1 and 2 [mean (SD) Likert-scale score 3.7 (0.48) vs 2 (1.3); *P* = 0.007].

One patient in the Group 2 had a small bowel injury (serosal tear), which was managed by primary repair.

The postoperative visual analogue scale (VAS) pain scores were compared between the two groups and at 8 h after surgery the Pfannenstiel-incision group (Group 2) reported less pain than the Iliac fossa-incision group (Group 1) [mean (SD) VAS pain score 3.14 (0.65) vs 2.85 (0.62), *P* = 0.018 at 8 h; 2.87 (0.64) vs 2.44 (0.71), *P* = 0.002 at 16 h; 2.19 (0.91) vs 1.85 (0.68), *P* = 0.03 at 24 h]. The Pfannenstiel-incision group required less postoperative analgesia compared to iliac fossa-incision group, at a mean (SD) of 7.03 (8.82) vs 15.55 (11.1) mg nalbuphine; *P* < 0.001. None of the patients in either group developed any wound-related complications. The mean haemoglobin drop and hospital stay was comparable between the groups (*P* = 0.85 and *P* = 0.24, respectively).

**Figure 4.**
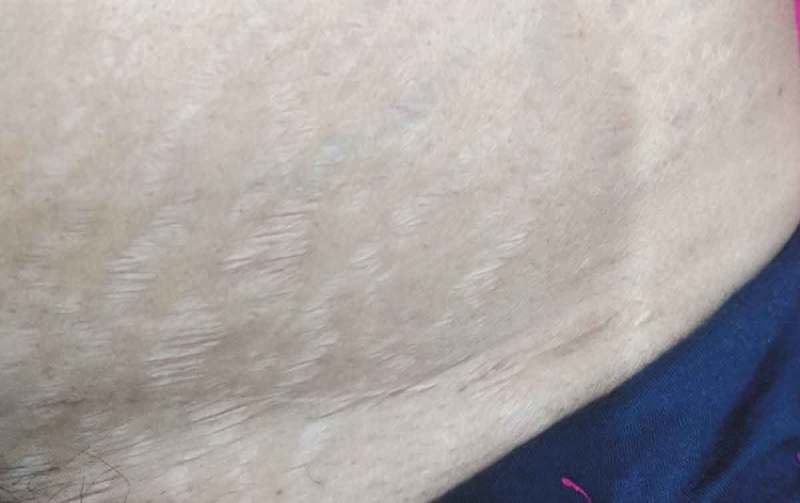
Iliac fossa incision scar at 6 months.

**Figure 5.**
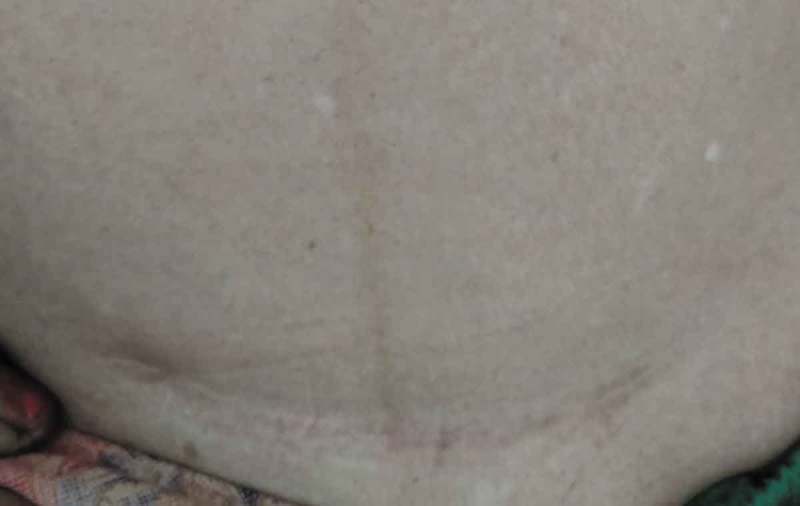
Pfannenstiel incision scar at 6 months.

During follow-up at 1 and 6 months, the Pfannenstiel-incision group had better Manchester Scar scores compared to the iliac fossa-incision group [mean (SD) 12.93 (0.84) vs 11.81 (0.8), *P* < 0.001; 7.83 (0.81) vs 6.63 (0.81), *P* < 0.001, respectively) (Figures 4 and 5).

## Discussion

The choice and placement of the retrieval incision play a critical role in the decision making of an individual to commit himself/herself to LDN, as the retrieval incision scar is the largest scar the patient will incur.

Through our prospective randomised study and the subsequent data evaluation, we tried to analyse the impact of a Pfannenstiel incision and iliac fossa incision on the ease of surgery, and graft- and patient-related outcomes.

In our present study, WIT was comparable in both the groups, at a mean (SD) of 310.37 (115.44) vs 310.65 (104.36) s (*P* = 0.99). This finding concurs with the finding in the Gupta et al. [10] study (3 vs 3.5 min, *P* = 0.18). However, in the Adiyat et al. [3] study, the WIT was significantly less in the Pfannenstiel-incision arm as compared to the iliac fossa-incision and midline-incision arms [mean (SD) 175 (59) vs 241 (62) vs 206 (49) s; *P* < 0.001]. In our present study, we additionally recorded the retrieval time, which was defined as the time from placement of first Hem-o-lok® clips (Weck Closure Systems, Research Triangle Park, NC, USA) on the renal artery until the time the kidney was extracted and placed on the ice slush. The retrieval time, which includes the WIT, was comparable in both the groups (*P* = 0.83). The retrieval time was not recorded in other comparative studies in the literature.

The mean (SD) total operative time in our present study was significantly different between the two groups, at 171.67 (36.81) vs 155.2 (27.77) min (*P* = 0.01), with Group 2 requiring less operative time than Group 1. This can be explained by the fact that the iliac fossa incision, being a muscle-cutting incision, requires more time during closure to ensure meticulous anatomical closure of all the anterior abdominal wall muscles. Whereas with a Pfannenstiel incision, the rectus abdominis muscles are retracted once the anterior rectus sheath is incised, thus requiring less time to approximate the muscles and the sheath. Gupta et al. [10] and Adiyat et al. [3] in their respective studies did not find a significant difference in mean operating time. Binsaleh et al. [11] compared the Pfannenstiel incision with an extended port-site incision in laparoscopic radical nephrectomy and found no statistically significant difference in operative time. However, Iemsupakkul et al. [12] compared Pfannenstiel vs extended iliac port-site kidney extraction in LDN and found a significant difference in operating time favouring the extended iliac port-site incision.

The true morbidity due to pain and subsequent analgesia requirement can be observed once the effect of anaesthesia wears off. In this phase, the Pfannenstiel incision scored better than the iliac fossa incision. Patients in the Pfannenstiel-incision group had better VAS pain scores at 8 h (mean (SD) 3.14 (0.65) vs 2.85 (0.62); *P* = 0.018], 16 h [2.87 (0.64) vs 2.44 (0.71); *P* = 0.002], and 24 h [2.19 (0.91) vs 1.85 (0.68); *P* = 0.03).

One of the main aspects of our present study was to focus our attention towards the assessment of intraoperative variables that might help us in making a choice of retrieval incision. For this purpose, a 5-point Likert scale-based surgeon’s assessment pro forma was handed to the operating surgeon after the procedure. A total of five parameters were assessed and scores for each parameter were compared between the two groups. The higher the score, the more the surgeon disagrees with the question at hand and the lesser the score the more the surgeon agrees with the statement. These parameters, to the best of our knowledge, were not addressed in the other studies comparing graft retrieval incision.

The first parameter (ease of placement of the incision) was found to have comparable scores in both the groups [mean (SD) Likert-scale score 2.2 (0.68) vs 2.22 (0.66); *P* = 0.88]. Gupta et al. [10] criticised the Pfannenstiel incision in their study, as the surgeon had to bend down to place the incision, which made it the less favoured incision in comparison to the iliac fossa incision. In our present study, the retrieval incision is placed once the patient is in the lateral decubitus position and strapped to the table. In the Pfannenstiel-incision group, the surgeon sits on a chair and then places the incision making it technically easy and less strenuous on the surgeons’ posture. The iliac fossa incision, due to its location can be easily placed in the standing positon and the surgeon did not appear to have any problem in placing this incision. Hence, the scores for this parameter were comparable in both groups. These finding was consistent in all the three BMI groups (Table 3).

The second parameter (interference of abdominal pannus in placing the incision) did show a significant difference in scores amongst the two groups [mean (SD) Likert-scale score 3.72 (0.76) vs 2.98 (1.36); *P* = 0.001). The interpretation of this result is that the abdominal pannus did interfere in placing the incision in the Pfannenstiel-incision group as compared to iliac fossa-incision group. As most of the donors in the Indian population have central obesity rather than generalised obesity, in the lateral decubitus position it is natural for the fat around the waist to fall towards the centre under gravity; thus, interfering with the placement of midline incisions like the Pfannenstiel incision. Not surprisingly, scores for this parameter were comparable in the group of donors with a BMI <25 kg/m^2^ (*P* = 0.35). However, in the group of donors whose BMI was 25–30 and >30 kg/m^2^, the difference in scores was statistically significant [mean (SD) Likert-scale score 3.48 (0.75) vs 2.16 (0.89), *P* < 0.001; and 3.7 (0.48) vs 2 (1.3), *P* = 0.007, respectively] (Table 3). The greater the BMI, the thicker the fat layer encountered during entry, as well as the exit of the surgeon’s hand, making these particular steps less easy as compared to when performed in a patient with a lesser BMI.

The third parameter (ease of specimen retrieval) showed a significant difference favouring the Pfannenstiel-incision group (*P* = 0.015). This can be explained by the difference in the locations of the two incisions. For retrieval of the kidney through the iliac fossa incision, the surgeon needs to elevate his shoulder, flex the forearm and medially rotate and then insert the hand inside, which is ergonomically a bit uncomfortable. Whereas, whilst retrieving the kidney through the Pfannenstiel incision, the incision being at a more caudal level than the iliac fossa incision does not demand the surgeon to elevate his/her shoulder. The surgeon needs just to bend a bit and insert his hand, which makes it ergonomically more appealing. For this parameter, the Pfannenstiel incision scored significantly better than the iliac fossa incision, especially in donors with a BMI <25 kg/m^2^ (*P* = 0.028). Whereas, in donors with a BMI of 25–30 (*P* = 0.09) and >30 kg/m^2^ (*P* = 0.73), BMI did not have a significant impact on the ease of specimen retrieval (Table 3).

For the very same above mentioned reasons, the Pfannenstiel incision provides a quick access to the hilar region and thus allows the surgeon to tackle any hilar bleeding during or immediately after the extraction of the kidney.

In the analysis of the fourth parameter (ease of immediate check laparoscopy), the Pfannenstiel incision scored better than the iliac fossa incision (*P* = 0.002). Check laparoscopy is done immediately after specimen retrieval, with the surgeon re-inserting his hand inside the abdomen under constant laparoscopic vision and gently dabbing the renal bed with a sterile mop. Mopping the renal bed is strictly discouraged by the authors, as it increases the risk of bleeding and dislodgement of the clips/Hem-o-lok. This is usually done to remove any large tenacious blood clots and tissue bits in the renal bed that would otherwise be difficult or time-consuming to remove laparoscopically. The other reason for an immediate check laparoscopy is to provide manual compression over the renal bed with a sterile mop to control small oozes and bleeders in the dissection area.

One possible explanation for the Pfannenstiel incision scoring better than the iliac fossa incision in this aspect is the fact that in the Pfannenstiel incision the rectus abdominis muscles that are retracted at the time of retrieval do have some amount of inherent tone under the effect of anaesthesia. When the surgeon inserts his/her hand inside the abdomen, the muscles tend to coil back and snugly hug around the surgeon’s forearm and thus contribute in creating an effective pneumoperitoneum. This is not the case with the iliac fossa incision where once the muscles are cut they lose their inherent tone and might not form a perfect airtight seal with the surgeon’s forearm. This impact of the retrieval incision on ease of immediate check laparoscopy was seen especially in donors with a BMI <25 kg/m^2^ (*P* = 0.012; Table 3).

The fifth parameter (ease of closure of the incision) was also analysed and here too the Pfannenstiel incision was better than the iliac fossa incision (*P* < 0.001). This can be explained by the very attribute of the Pfannenstiel incision being a muscle-splitting one and the iliac fossa incision being a muscle-cutting incision. In the iliac fossa incision, it has been observed that while placing the incision, once the muscles are cut, they tend to retract beneath the skin and subcutaneous tissue. This makes the identification of the layers of muscles a bit challenging during the time of closure. This in turn is reflected in the difficult and time-consuming muscle approximation. Whereas, while placing the Pfannenstiel incision, only the anterior rectus sheath is incised and the rectus abdominis muscles are retracted from the linea alba. At the time of closure, the rectus abdominis muscles tend to fall back to their respective anatomical positons making their approximation quite easy and quick. Additionally, the anterior rectus sheath that was incised is easily identifiable, as it tends to remain in place and not retract vis-à-vis the iliac fossa muscles. Both these factors seem to contribute in the ease of closure of the retrieval incision in the Pfannenstiel-incision group. Having said that, this advantage of the Pfannenstiel incision is lost in obese patients, as in our present study, as the BMI increased over 30 kg/m^2^, both the incisions were comparable in this aspect (*P* = 0.33; Table 3)

During the course of the present study, two patients in the Pfannenstiel-incision arm were excluded from the study because of pelvic adhesions that hindered the performance in a safe way and required a change to an iliac fossa incision for retrieval. This represents 3.7% of the whole cohort. The reason for the development of these adhesions was unknown. Thus, the authors strongly advise a thorough check laparoscopy before the commencement of surgery to be very sure of the choice of retrieval incision.

Our present study showed that during follow-up, neither of the incisions was associated with any postoperative wound complications. However, the Pfannenstiel incision demonstrated a satisfactory cosmetic outcome as suggested by the Manchester Scar scores [mean (SD) at 1 month, 12.93 (0.84) vs 11.81 (0.8); *P* < 0.001; and at 6 months, 7.83 (0.81) vs 6.63 (0.81); *P* < 0.001). Gupta et al. [10], in their study also showed similar findings with the Pfannenstiel incision having a better cosmetic outcome than the iliac fossa incision.

### Study limitations

The authors of the study do acknowledge that even though BMI was one of the variables, the impact of which was studied on the outcome of the two incisions, it was not comparable between the two groups at point of entry of patients into the study. The two groups under study were not comparable in terms of BMI as the difference in BMI was significant between the Group 1 and Group 2, at a mean (SD) of 26.79 (4.14) vs 25.26 (3.55) (*P =* 0.04).

## Conclusion

The Pfannenstiel incision was considered preferable during the critical steps of the LDN, e.g., specimen retrieval, immediate check laparoscopy, and incision closure. The Pfannenstiel incision was associated with a smaller retrieval incision, lesser operative time, less postoperative pain and analgesia requirement compared with the iliac fossa incision. Our present study showed that during follow-up, neither of the incisions was associated with any postoperative wound complications and the Pfannenstiel incision had a satisfactory cosmetic outcome.
